# A CT-based radiomics approach to predict immediate response of radiofrequency ablation in colorectal cancer lung metastases

**DOI:** 10.3389/fonc.2023.1107026

**Published:** 2023-01-31

**Authors:** Haozhe Huang, Dezhong Zheng, Hong Chen, Chao Chen, Ying Wang, Lichao Xu, Yaohui Wang, Xinhong He, Yuanyuan Yang, Wentao Li

**Affiliations:** ^1^ Department of Interventional Radiology, Fudan University Shanghai Cancer Center, Shanghai, China; ^2^ Department of Oncology, Shanghai Medical College, Fudan University, Shanghai, China; ^3^ Laboratory for Medical Imaging Informatics, Shanghai Institute of Technical Physics, Shanghai, China; ^4^ Department of Electronic, Electrical and Communication Engineering, University of Chinese Academy of Sciences, Beijing, China; ^5^ Department of Medical Imaging, Shanghai Mental Health Center, Shanghai Jiao Tong University School of Medicine, Shanghai, China

**Keywords:** computed tomography (CT), radiomics, clinical variables, colorectal cancer, lung metastasis, radiofrequency ablation (RFA), efficacy evaluation

## Abstract

**Objectives:**

To objectively and accurately assess the immediate efficacy of radiofrequency ablation (RFA) on colorectal cancer (CRC) lung metastases, the novel multimodal data fusion model based on radiomics features and clinical variables was developed.

**Methods:**

This case-control single-center retrospective study included 479 lung metastases treated with RFA in 198 CRC patients. Clinical and radiological data before and intraoperative computed tomography (CT) scans were retrieved. The relative radiomics features were extracted from pre- and immediate post-RFA CT scans by maximum relevance and minimum redundancy algorithm (MRMRA). The Gaussian mixture model (GMM) was used to divide the data of the training dataset and testing dataset. In the process of modeling in the training set, radiomics model, clinical model and fusion model were built based on a random forest classifier. Finally, verification was carried out on an independent test dataset. The receiver operating characteristic curves (ROC) were drawn based on the obtained predicted scores, and the corresponding area under ROC curve (AUC), accuracy, sensitivity, and specificity were calculated and compared.

**Results:**

Among the 479 pulmonary metastases, 379 had complete response (CR) ablation and 100 had incomplete response ablation. Three hundred eighty-six lesions were selected to construct a training dataset and 93 lesions to construct a testing dataset. The multivariate logistic regression analysis revealed cancer antigen 19-9 (CA19-9, p<0.001) and the location of the metastases (p< 0.05) as independent risk factors. Significant correlations were observed between complete ablation and 9 radiomics features. The best prediction performance was achieved with the proposed multimodal data fusion model integrating radiomic features and clinical variables with the highest accuracy (82.6%), AUC value (0.921), sensitivity (80.3%), and specificity (81.4%).

**Conclusion:**

This novel multimodal data fusion model was demonstrated efficient for immediate efficacy evaluation after RFA for CRC lung metastases, which could benefit necessary complementary treatment.

## Introduction

Colorectal cancer (CRC) is one of the most common malignant tumors and a leading cause of cancer-related mortality worldwide (1). About 25% of CRC patients present with distant metastases at the time of initial diagnosis, with the most common sites including liver and lung (2, 3). In addition, patients with rectal cancer are more likely to have lung metastases because of anatomical differences (4, 5). However, not all patients meet the criteria for surgical resection due to lesion location, tumor burden, comorbidity, or the presence of extra-pulmonary disease. For this group, thermal ablation, including radiofrequency (RFA) or microwave (MWA), is considered a safe alternative (6).

RFA has been proven safety and efficacy in lung metastases from CRC (7–9). However, there is no pathological histological evidence of complete ablation after RFA, and recent studies demonstrated that the incomplete RFA promoted increased tumorigenesis (10) and hindered the efficacy of anti-programmed cell death protein-1 immunotherapy (11). In addition, the existence of remnant tumor masses was associated with earlier new metastases and poor survival (11). Therefore, it is crucial to clarify the local recurrence factors and assess the early-stage efficacy. To achieve complete ablation of lung cancer, any peritumoral lung parenchyma within 5 to 10 mm needs to be ablated (12–14). This area presents as necrosis, effusion and congestion from the inner zone to the outer zone on histopathology, accordingly (15), and manifests as ground-glass opacity (GGO) on CT, which is the typical post-ablation presentation and the crucial area in the early assessment after RFA (16). Previous studies based on the morphological changes of unenhanced CT found that the size of GGO was associated with residual tumor and recurrence (17, 18). However, intraoperative complications such as intra-alveolar hemorrhage (IAH) or atelectasis, make it impossible to determine the extent of ablation (9, 12, 19, 20). Therefore, the observation and measurement of the intraoperative GGO range to ascertain whether ablation is complete is subjective and uncertain as such an approach is easily influenced by doctors with differences experience.

The modified response evaluation criteria in solid tumors (mRECIST) are used to evaluate the efficacy of lung tumor ablation (21–23). However, the inflammatory response surrounding the lesion make it difficult to clearly evaluate the early efficacy. The lesions do not stabilize or shrink until at least six months after ablation, eventually manifesting in the form of disappearance, fibrosis, nodules, and cavities (24, 25). This time-lapse evaluation method may also result in a missed opportunity for the optimal complementary therapy for patients, thus affecting their survival benefits. Therefore, there is an urgent need for objective and reliable characteristic metrics or models to evaluate the immediate ablative efficacy of RFA for pulmonary metastases.

Radiomics can mine high-dimensional quantitative imaging features of medical images, which contain information related to tumor heterogeneity and microenvironment (26–29), allowing for more accurate quantification of phenotypic features and assessment of treatment response (30–33). Radiomics analysis includes target lesion segmentation, feature extraction, machine learning classifier training, and performance evaluation (34–36). However, the radiomics feature analysis approach just takes full advantage of a single mode of radiological data which is incomplete and noisy whilst ignoring other modalities data, such as histopathology, genomics, or clinical information, leaving multimodal data integration relatively underdeveloped (37).

In this study, we developed novel multimodal data fusion models integrating radiomics features based on radiological data with clinical variables originating from textual data to assist interventional physicians in evaluating the immediate efficacy of RFA for CRC lung metastases, so as to make necessary supplementary treatment during operation.

## Materials and methods

### Data collection

**Figure 1:**
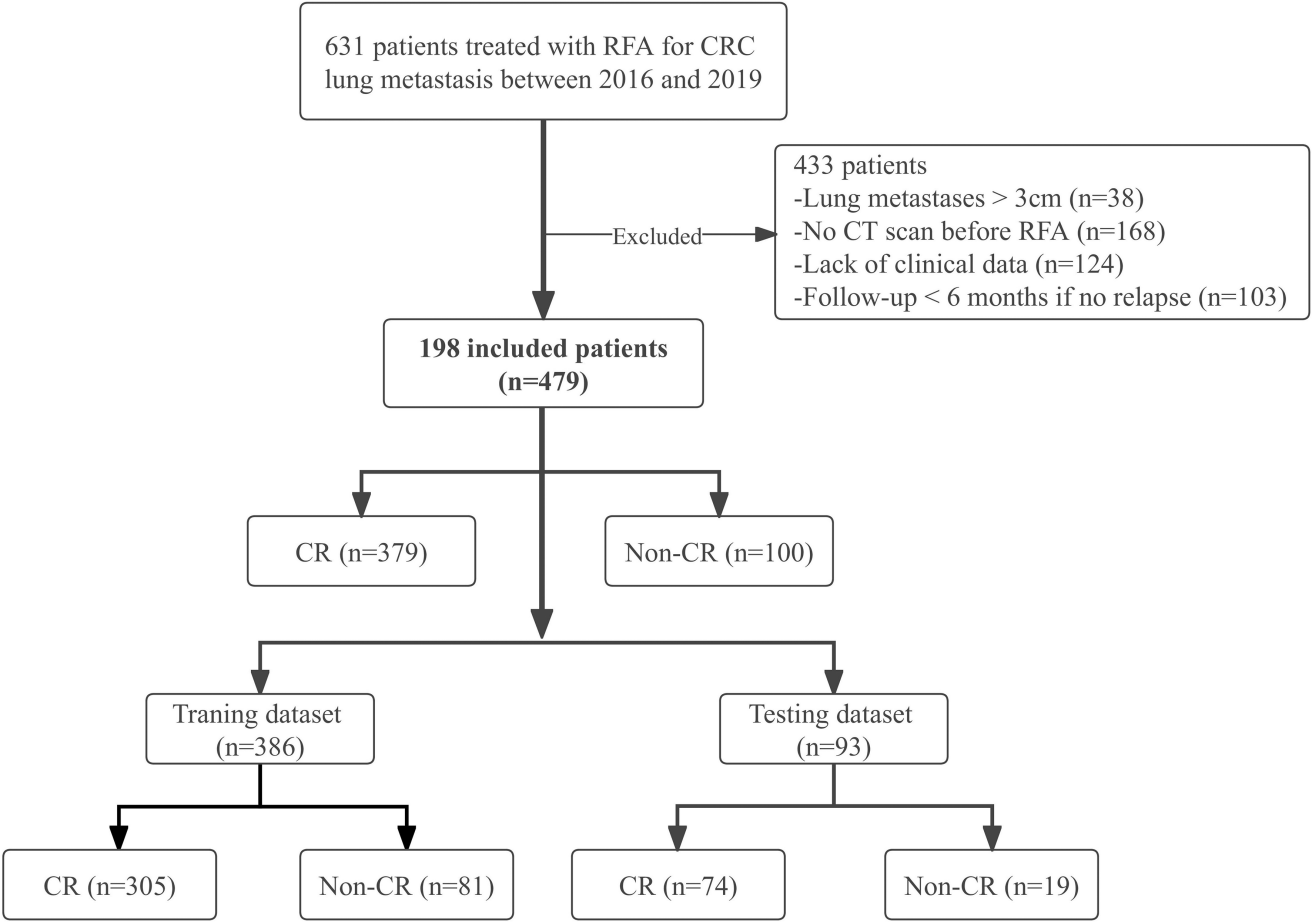
Study flow chart. CRC, colorectal cancer; CR, complete response; Non-CR, Non-complete response.

CRC patients with lung metastases who underwent percutaneous RFA under CT guidance between August 2016 and January 2019 were enrolled in this study. Patients were recruited based on the following eligibility criteria: (1) histologically confirmed CRC; (2) ablated lung metastases with maximum diameter ≤3 cm; (3) chest enhanced CT examination within 4-6 weeks before RFA; (4) complete CT images during the procedure; (5) re-examination by chest enhanced CT at least 6 months after RFA; (6) technically successful ablation; (7) adequate normal organ function. Exclusion criteria, based on the European Society for Medical Oncology (ESMO) guidelines (38) were: (1) > 5 lung metastases; (2) maximum diameter > 3 cm; (3) other local or regional treatments such as radiotherapy before or after RFA; (4) incomplete clinical data; (5) second ablation (i.e., re-ablation). We allowed the inclusion of patients with multiple nodules and analyzed each nodule individually. A cohort of 198 patients with 479 lung metastases who received RFA was retrospectively selected (**Figure 1**).

The following clinical data were retrieved: age at diagnosis, gender, serum tumor markers, including carcinoembryonic antigen (CEA) and cancer antigen 19-9 (CA19-9). The radiological data were recorded as follows: the location of pulmonary metastases, proximity to the heart, great blood vessels (diameter > 3 mm), pleura or diaphragm (within 1 cm) through the preoperative CT images; the IAH or pneumothorax were acquired.

All CT examinations (United Imaging uCT 760, Shanghai United Imaging Medical Technology Inc., China and Philips Brilliance 64 slice, Philips Medical Systems Inc., USA) were performed with a fixed tube current of 200 mA and a tube voltage of 120 kVp. The pixel spacing ranged from 0.684 to 0.748 mm, and the slice thickness was 1 mm or 1.5 mm. The intraoperative CT images were of fixed tube current of 200 mA, tube voltage of 120 kVp, and slice thickness of 1 mm or 3 mm. The image reconstruction method of both CT scanners is iterative reconstruction. Radiological follow-up consisted of chest-enhanced CT scans performed at 1, 3, 6, 12 months, and every 6 months after that. The shortest follow-up time was over 6 months.

This study was approved by the Institutional Review Board of the Ethics Committee of Fudan University Shanghai Cancer Center. Written informed consent was obtained from all patients.

### RFA procedures and local efficacy assessment

RFA mainly utilizes 460 ~ 480 kHz high-frequency current to heat a tissue volume around a needle electrode and induce focal coagulative necrosis with minimal injury to surrounding tissues (39, 40). Here, RFA was performed using a radiofrequency applicator (MedSphere International), with the mode of temperature control or impedance control for choice. The power settings were adjusted according to the manufacturer’s protocols: 5 min for a 2.0 ~ 2.5 cm active tip at 30 W, 8 min for a 3.0 ~ 3.7 cm active tip at 50 W, and 10 min for a 4.0 ~ 4.7 cm active tip at 60 W, respectively.

All the operations were performed by three senior interventional radiologists (L.X., Y.W. and X.H. with over 10 years of experience in thoracic interventions under CT guidance). Depending on the location of the target nodules, patients were placed in a prone position, lateral position or supine position to ensure the best puncture site and entry route and avoid important structures, including ribs, interlobular fissures, and blood vessels. Lidocaine was administered at the puncture site to induce local anesthesia of the pleura. With CT monitoring, the radiofrequency electrode was punctured according to the predetermined direction and angle. The ablation was not performed until the CT scan confirmed that the electrode hooked the lesion. Considering tumor shape and size, one or two needle ablations with a constant antenna position were usually acceptable to achieve complete ablation. The operators strived to achieve ablation range greater than the lesions by at least 5 mm. If the intraoperative complications such as intra-alveolar hemorrhage (IAH) or atelectasis, made it impossible to determine the extent of ablation, at least 2 cycles of ablation would be performed to raise the impedance until ablation stopped. After completion of the RFA session, the ablation electrode was withdrawn, and a repeat CT (same parameters) scan was performed to evaluate whether the ablation zones covered the tumor and the occurrence of ablation-related complications, mainly including pneumothorax and hemorrhage.

Local efficacy was assessed by two radiologists who were blind to clinical data (H.C. and H.H. with over 5 years of experience) through chest enhanced CT examination at least 6 months after RFA according to mRECIST criteria (24, 41). If they had disagreements, it would be determined in consultation with the senior expert (W.L. with over 20 years of experience). The follow-up CT examination one month after ablation was taken as the baseline (42). Based on the mRECIST criteria, CR was defined if any of the following manifestations on CT were seen: the disappearance of the lesion, cavity, fibrosis or nodule without enhancement. If two consecutive CT examinations demonstrated the target lesions had irregular enlargement or enhanced solid components, they were classified as a non-complete response (non-CR).

### Pre-processing of CT images, radiomics feature extraction, selection and data division

**Figure 2:**
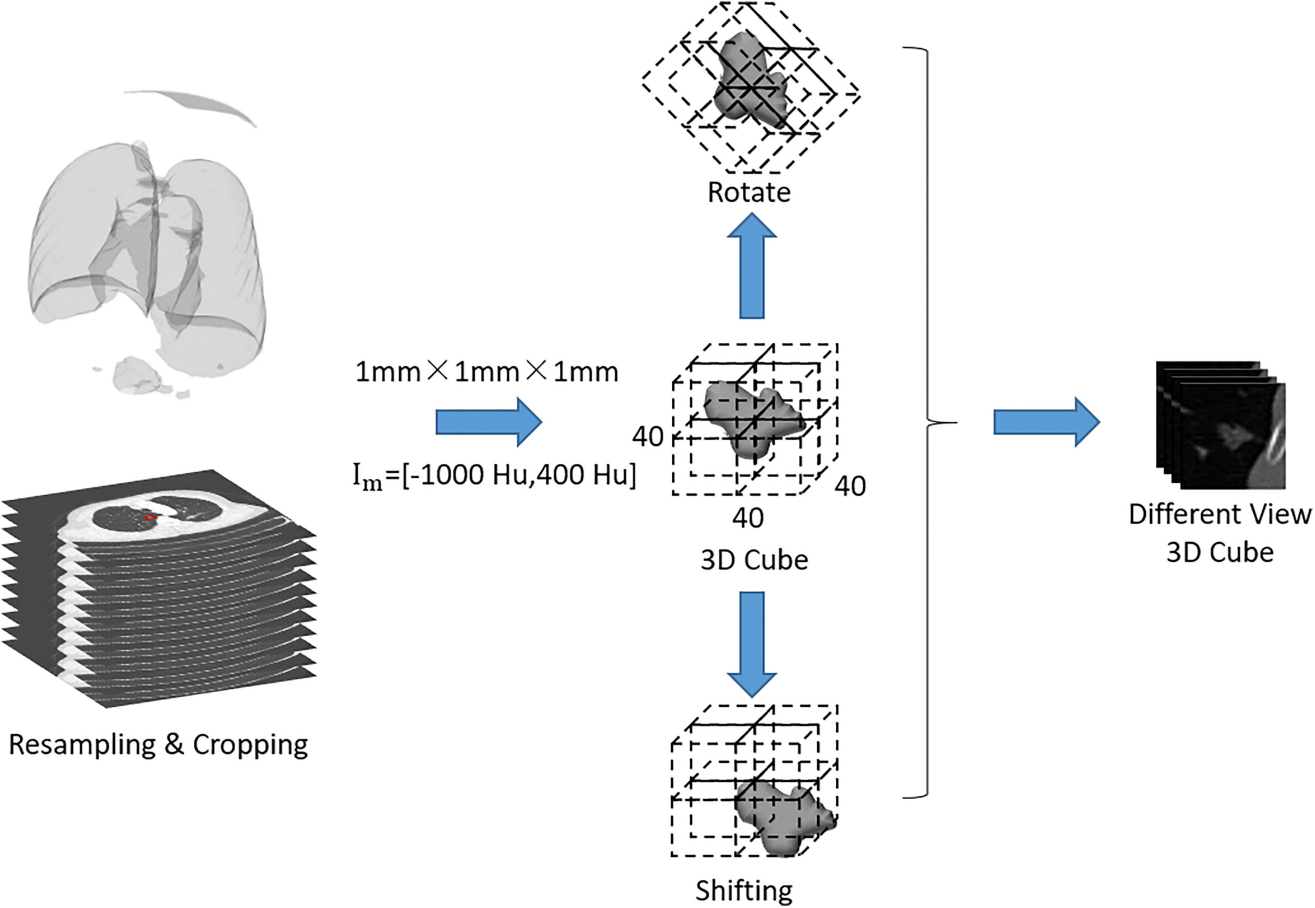
Flow chart of image preprocessing.

In order to avoid the data bias due to the difference in scanning spacing and slice thickness between preoperative and immediately postoperative CT images, the following preprocessing steps were adopted: the CT images were uniformed to a common resolution of 1 mm × 1 mm × 1 mm by B-spline interpolation algorithm, and then the window width was adjusted within the range of - 1200 Hu to 600 Hu and the intensity was scaled within the range of 0 ~ 255. After normalization of all CT images, the samples containing pulmonary metastases were trimmed to 3D cubes with the size of 40 mm × 40 mm × 40 mm. Finally, the gray values of sample cubes were normalized between 0 and 1 (**Figure 2**).

**Figure 3:**
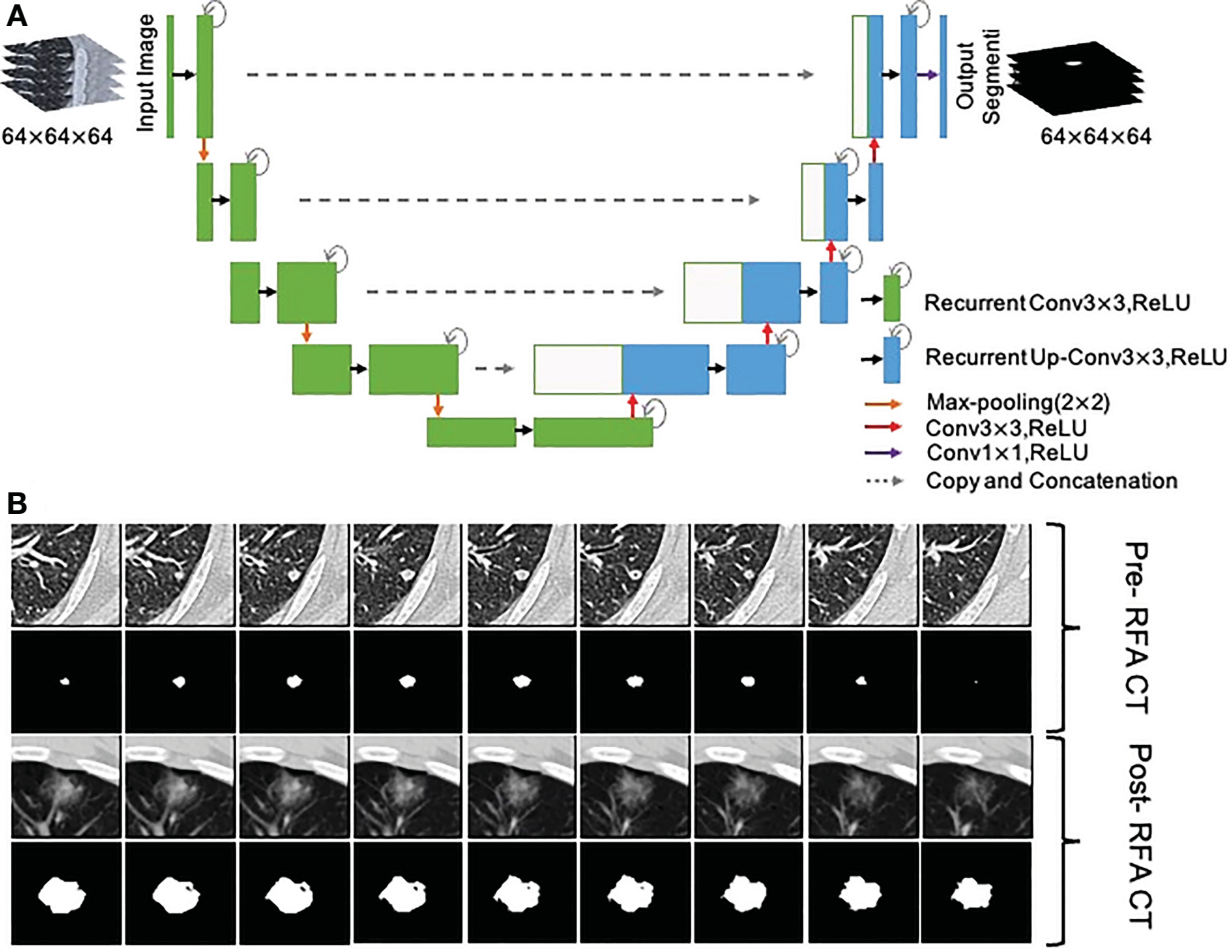
Segmentation of metastasis and ablation area. **(A)** Flow chart of the 3D U-Net model; **(B)** CT images and segmentation images of colorectal cancer lung metastasis and ablation area immediate after RFA.

To objectively and accurately delineate the target lesions in the preoperative CT images and the boundary of the ablation area immediately after RFA, A 3D U-Net model (43) was used to segment the lesions and ablation region automatically, and two junior radiologists (H.C.and H.H.) verified the segmentations and made the necessary adjustments to guarantee the accurancy and repeatability (**Figure 3**). If they had disagreements, it would be determined in consultation with the senior expert (W.L.).

For each segmented preoperative lesions and ablation region, 1252 radiomics features were extracted through the open-source feature toolboxes PyRadiomics (44) and PREDICT. The radiomics features were comprised of 13 intensity features, 35 shape features, 9 orientation features and 507 texture features which contained 144 Gray Level Co-occurence Matrix (GLCM) features, 16 Gray Level Size Zone Matrix (GLSZM) features, 16 Gray Level Run Length Matrix (GLRLM) features, 14 Gray Level Dependence Matrix (GLDM) features, 5 Neighborhood Grey Tone Difference Matrix (NGTDM) features, 156 Gabor filters features, 39 Laplacian of Gaussian (LoG) filters features, 39 Local Binary Patterns (LBP) features (32, 44–47), and 688 wavelet features.

In order to reduce unnecessary, redundant information and complexity in the process of calculation and modeling, the maximum correlation and minimum redundancy algorithm (MRMRA) (48) was used for features selection. There are five common variants under the MRMRA framework (49): mutual information difference (MID), mutual information quotient (MIQ), F-test correlation difference (FCD), F-test correlation quotient (FCQ), and random-forest correlation quotient (RFCQ). The formulas were as follows:

Assuming that there were m features in total, for a given feature Xi, i∈ (1, 2,…, m), the importance of the feature could be determined by MRMRA, commonly in the following five forms:


$$f^{MID}(X_{i})=I(Y,X_{i})-\frac{1}{\left|S\right|}\sum X_{s}\in SI(X_{s},X_{i})$$

$$f^{MIQ}(X_{i})=I(Y,X_{i})/[\frac{1}{\left|S\right|}\sum X_{s}\in SI(X_{S},X_{i})]$$

$$f^{FCD}(X_{i})=F(Y,X_{i})-\frac{1}{\left|S\right|}\sum X_{S}\in SP\left(X_{S},X_{i}\right)$$

$$f^{FCQ}(X_{i})=F(Y,X_{i})/[\frac{1}{\left|S\right|}\sum X_{S}\in SP(X_{S},X_{i})]$$

$$f^{RCQ}(X_{i})=I_{RF}(Y,X_{i})/[\frac{1}{\left|S\right|}\sum X_{S}\in SP(X_{S},X_{i})]$$

where, *Y* is the category label corresponding to the variable, *S* is the selected feature set, |*S*| is the size of the feature set, *Xs*∈*S* is a feature outside the feature set *S*, and *Xi* represents a feature that was not currently selected; the function*I*(·,·) represents mutual information,*p*(·,·) is the Pearson correlation coefficient, *F*(·,·) is the *F*statistics, and *I*_*RF*_(·,·) is the random forest feature importance score. Since inconsistent results of various methods under different super parameter conditions, we utilized the above 5 methods to filter features. The frequency of the top 5, top 10, and top 15 features was counted in the importance ranking, and the experiments were conducted from 5 to 15 features with the highest frequency to obtain the best performance, and eventually to confirm the 9 selected features.

As the Gaussian mixture model (GMM) had good performance in the evaluation of sample distribution and similarity in high-dimensional space (50–52), we used distance metric learning based on the Gaussian mixed model (DML-GMM) rather than random splitting to divide data according to our previous research results (53). We demonstrated that when the sample size was large, there was little difference between random splitting and the DML-GMM model. As for a smaller sample size, however, the DML-GMM model could obtain more stable results than random splitting. Therefore, the log-likelihood of the extracted radiomic features was calculated by DML-GMM model to describe the distribution, the data was split into multiple clusters and then was divided into 5 groups by stratified sampling. One group was selected as the testing set and the remaining 4 groups were used as the training set. Actually, we did five-fold cross-validation and chose a single split data including 386 lesions for the training set and 93 for the testing set.

### Model building and performance evaluation

Due to the unbalanced distribution of case counts in CR and non-CR, we adopted the oversampling method (synthetic minority over-sampling technique, SMOTE) (54) to mitigate the biased impact of data imbalance on the models during training.

Clinical model: all the clinical and radiological features were included in the univariate Logistic regression analysis, after which the variables with P< 0.1 were included in the multivariate analysis. Finally, the independent factors with P< 0.05 were selected for modeling. The random forest technique was a regression tree technique which utilized bootstrap aggregation and randomization of predictors to achieve a high degree of predictive accuracy (55). Since the random forest algorithm has been proven to be effective and superior in building clinical models (56, 57), our clinical model was also built on it.

**Figure 4:**
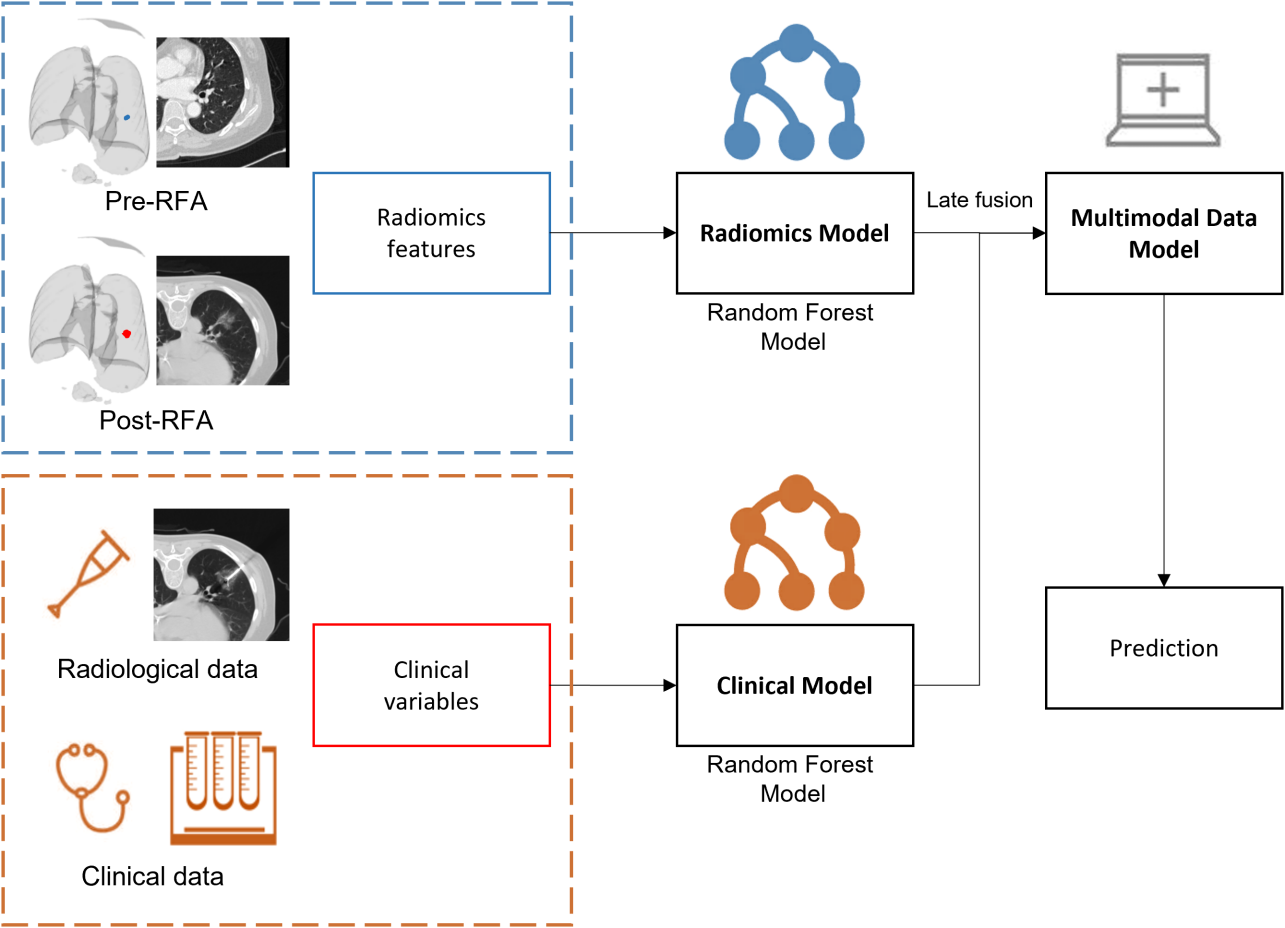
Fusion framework of radiomics features and clinical information.

Radiomics model: A random forest, which was the most common classifier used for radiomics features classification, contained multiple decision trees, and the total output result was determined by the subcategories of each decision tree. When processing high-dimensional data, it had a strong ability of anti-interference and anti-overfitting, especially for unbalanced medical data. Several studies have confirmed that the random forest model could be used to predict the survival rate, recurrence risk, and efficacy evaluation of lung cancer patients (58–60).Multimodal data fusion models: the random forest model was integrated based on radiomics and clinical models (**Figure 4**). The weighted fusion strategy (61) adopted in our study was decision level fusion (late fusion) (62). This level of fusion allowed features from different representations to be combined in the same format of representation, which had more and better scalability and flexibility (63). The exact formula was *confidence*=*ω*_1_·*confidence*_*image*_+*ω*_2_·*confidence*_*clinical*_· The fusion prediction score was calculated to obtain the final prediction result.

In order to evaluate the performance of various models, we validated them on an independent test dataset, drew receiver operating characteristic curves (ROC) with the obtained prediction scores and calculated the corresponding area under curve (AUC). The difference in the predictive performance of models was compared by the Delong test (64). Meanwhile, the accuracy $\left(\text{ACC}=\frac{\text{TP}+\text{TN}}{\text{TP}+\text{FP}+\text{TN}+\text{FN}}\right)$ , sensitivity $\left(\text{Sensitivity}=\;\frac{\text{TP}}{\text{TP}+\text{FN}}\right)$ , and specificity $\left(\text{Specificity}=\;\frac{\text{TN}}{\text{TN}+\text{FP}}\right)$ were also calculated. In the formula, TP was true positive, FP was false positive, TN was true negative and FN was false negative.

### Statistical analysis

Statistical analyses were performed using IBM SPSS (version 26.0, Chicago, USA). Man-Whitney U test was used for continuous variables which were presented as mean ± standard deviation (SD). Chi-square or Fisher test was used for categorical variables. All statistical tests were conducted at a two-sided significance level of P<0.05. All the medical image processing procedures and evaluation processes were performed on Python 3.6. In order to build the models and calculate the evaluation scores, we used publicly available packages such as SimpleITK, PyTorch, scikit-learn, numpy, and scipy.

## Results

### Characteristics of patients and lesions

A total of 198 patients with 479 lung metastases from CRC were enrolled; the detailed demographic characteristics are listed in **Table 1**. After RFA treatment, there were 379 CR lesions and 100 non-CR lesions. Due to the small sample size, we analyzed each lesion individually in the same patient with multiple metastases as the recent literatures (20, 65–67). Through the GMM method, 386 lesions (305 CR and 81 non-CR) were selected to constitute the training dataset, and 93 lesions (74 CR and 19 non-CR) were chosen to constitute the independent testing dataset (**Figure 1**). There were 227 lesions (47.4%)< 10 mm, and most lesions (399, 83.3%) were not close to the mediastinum or great vessels (diameter greater than 3 mm), but close to the pleura or diaphragm (287, 59.9%). The incidences of IAH and pneumothorax were 25.9% (124/479) and 24.0% (115/479), respectively.

**Table 1:** Characteristics of patients and colorectal cancer lung metastases.

Characteristics		Training dataset(N= 386)	Testing dataset(N=93)	P	Total(N=479)
**Pre-RFA clinical features**					
**Gender**	Male	221	48	0.0347	269
	Female	165	45		210
**Age (years, mean ± SD)**		57.9±10.3	57.3±11.1	0.5860	
**Tumor markers**	CEA (ng/ml)	4.6±4.1	3.9±3.6	0.4594	
	CA19-9 (U/ml)	10.8±6.2	10.9±6.4	0.8112	
**Pre-RFA characteristics of the lung metastasis**					
**Nodule size (mm)**	< 10	186	41	0.0622	227
	10–19	141	37		178
	20 - 30	59	15		74
**Location**	RUL	91	21	0.0011	112
	RML	41	11		52
	RLL	76	12		88
	LUL	72	36		108
	LLL	106	13		119
**Distance 1 (cm)**	> 1	329	70	0.3997	399
	< 1	57	23		80
**Distance 2 (cm)**	> 1	151	41	0.0739	192
	< 1	235	52		287
**Immediate post-RFA features**					
**Pneumothorax**	Yes	92	23	0.2932	115
	No	294	70		364
**Intra-alveolar**	Yes	100	24	0.2654	124
**Hemorrhage**	No	286	69		355

RUL, right upper lobe; RML, right middle lobe; RLL, right lower lobe; LUL, left upper lobe; LLL, left lower lobe; SD, standard deviation; Distance 1, the distance between the lesion and the large vessels or mediastinum; Distance 2, the distance between the lesion and the pleura or diaphragm.

### Clinical and radiomics feature selection

Univariate logistic regression analysis in **Table 2** showed that CEA, CA19-9, lesion location (including upper lobe of the right lung, right lower lobe, and left lower lobe), and intra-alveolar hemorrhage (P< 0.1) could completely identify ablated lesions. Furthermore, multivariate regression analysis demonstrated that CA19-9 (odds ratio [OR] = 1.007, P< 0.001) and lesion location (including right upper lobe [OR = 1, P = 0.005], right lower lobe [OR = 2.997, P = 0.003], and left lower lobe [OR = 2.498, P = 0.011]) were independent risk factors for incomplete ablation. These two clinical variables were used to construct a clinical model.

**Table 2:** Uni- and multi-variate analysis of clinical and radiological characteristics.

Characteristics			Univariate analysis		Multivariate analysis	
			OR (95% CI)	P	OR (95% CI)	P
Clinical features						
**Gender**	Male		1.259 (0.803-1.974)	0.316		
	Female		1			
**Age**			0.994 (0.974-1.015)	0.588		
**Tumor biomarkers**	CEA		1.007 (1.001-1.012)	**0.028**	1.004 (0.999-1.009)	0.166
	CA 19-9		1.006 (1.002-1.009)	**0.001**	1.007 (1.003-1.011)	**<0.001**
Pre-RFA features of the lung metastases						
**Location**		RUL	1	**0.013**	1	**0.005**
		RML	1.323 (0.538-3.253)	0.542	0.949 (0.357-2.525)	0.917
		RLL	2.968 (1.468-6.002)	**0.002**	2.997 (1.442-6.23)	**0.003**
		LUL	1.293 (0.615-2.718)	0.497	1.216 (0.557-2.654)	0.624
		LLL	2.23 (1.125-4.419)	**0.022**	2.498 (1.228-5.08)	**0.011**
**Distance 1 (cm)**		> 1	1			
		< 1	0.693 (0.402-1.197)	0.189		
**Distance 2 (cm)**		> 1	1			
		< 1	0.957 (0.608-1.506)	0.848		
Immediate post-RFA features						
**Pneumothorax**		Yes	1.08 (0.652-1.789)	0.764		
		No	1			
**IAH**		Yes	0.612 (0.354-1.059)	**0.079**	0.644 (0.364-1.138)	0.130
		No	1		1	

The bold p vales in the univariate analysis (in the first column) mean < 0.1, and those in the multivariate analysis (in the second column) mean < 0.05.

In order to prevent the model from overfitting because of the small sample size, 5 to 15 vital features with the highest scores were selected by MRMRA, and the five forms of MRMRA features importance scores were calculated separately, and compared with the default important feature s of the random forest model as the benchmark. The results demonstrated that the important features selected by MRMRA in the form of MID, MIQ, FCQ, and RFCQ had better performance than the features automatically selected by random forest, and the experimental model with 9 selected features had achieved better stability and smaller deviation. The selected feature results are shown in **Table 3**.

**Table 3:** Radiomics features selected by MRMRA.

pre-RFA radiomics features	post-RFA radiomics features
shape_Elongation	GLCM_Idmn
GLCM_Idmn	GLRLM_RunEntropy
GLCM_Imc1	GLCM_Imc2
GLCM_InverseVariance	
GLCM_ClusterShade	
GLDM_DependenceEntropy	

MRMRA, maximum relevance and minimum redundancy algorithm; RFA, radiofrequency ablation; GLCM, Gray Level Co-occurence Matrix; GLDM, Gray Level Dependence Matrix; GLRLM, Gray Level Run Length Matrix.

### Prediction performance comparison

**Figure 5:**
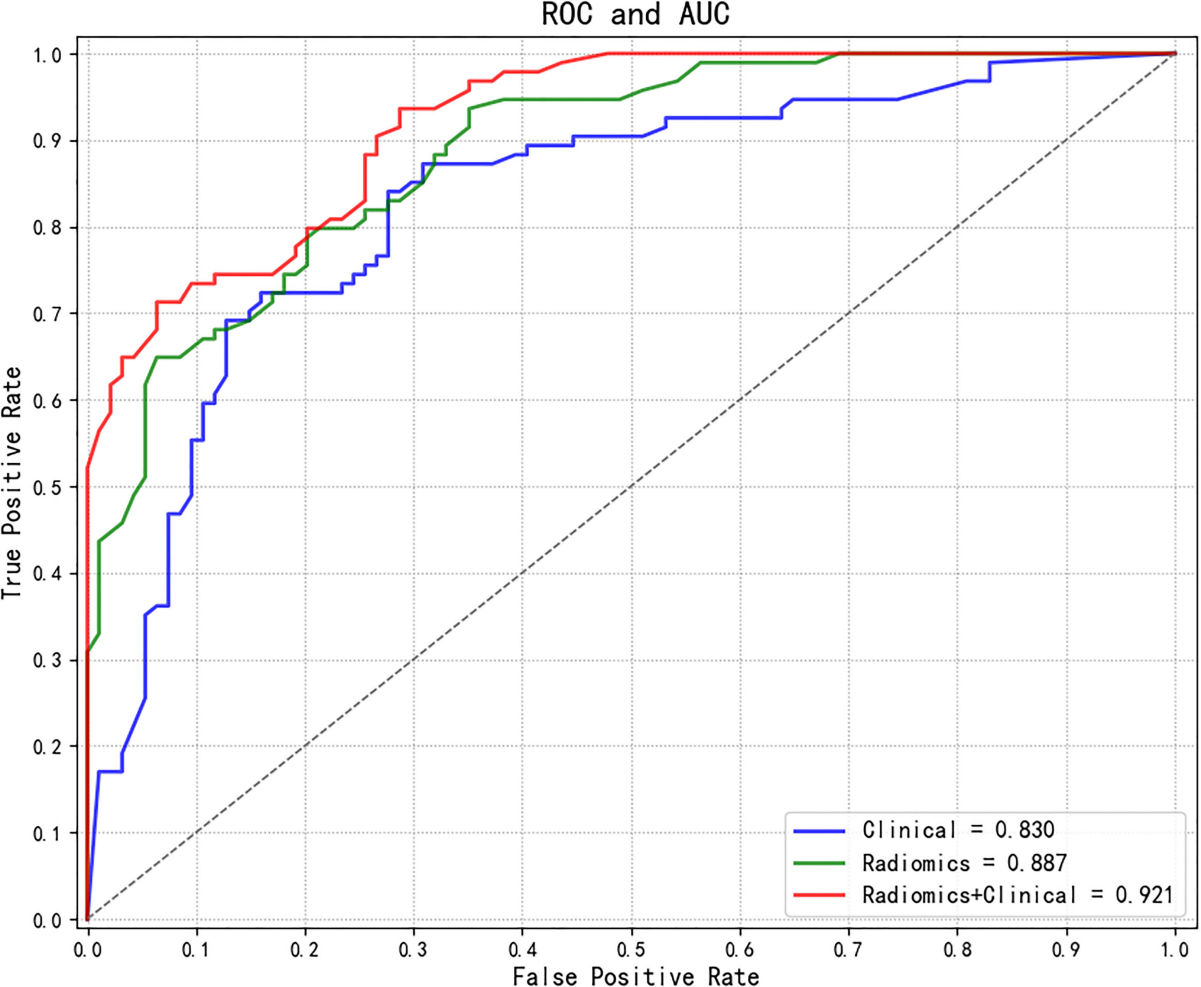
Comparisons of ROC curves of different models. ROC, receiver operating characteristic; AUC, area under the curve.

The AUC values of each model were calculated in an independent testing dataset, and the DeLong test compared the corresponding P values (**Tables 4**, **5** and **Figure 5**). When radiomics features were integrated with clinical variables, and the coefficient of the radiomics model was 0.7 and the coefficient of clinical model was 0.3, the resulting AUC value achieved the highest (0.921) with the statistically significant difference (P values of 0.043) compared with the clinical model alone (0.830). In addition, the accuracy, sensitivity, and specificity of this multimodal data fusion model were also the best (82.6%, 80.3%, and 81.4%, respectively).

**Table 4:** AUC values of different models in the testing dataset.

Models	AUC
**Clinical**	0.830
**Radiomics**	0.887
**Radiomics + clinical**	0.921
**0.1× Radiomics + 0.9 × clinical**	0.839
**0.2× Radiomics + 0.8 × clinical**	0.852
**0.3× Radiomics + 0.7 × clinical**	0.869
**0.4× Radiomics + 0.6 × clinical**	0.885
**0.5× Radiomics + 0.5 × clinical**	0.904
**0.6× Radiomics + 0.4 × clinical**	0.913
**0.7× Radiomics + 0.3 × clinical**	**0.921**
**0.8× Radiomics + 0.2 × clinical**	0.916
**0.9× Radiomics + 0.1 × clinical**	0.903

The bold value means the highest AUC value of the best model.

**Table 5:** Comparison of prediction performance of different models in the testing dataset.

Models	ACC (%)	AUC	Sensitivity (%)	Specificity (%)
Clinical	71.4	0.830	69.6	75.3
Radiomics	80.8	0.887	79.1	80.6
**Radiomics + Clinical**	**82.6**	**0.921**	**80.3**	**81.4**

ACC, accuracy; AUC, area under ROC curve.

The bold values mean the best performance of the multimodal data fusion model integrating radiomic features and clinical variables.

**Figure 6:**
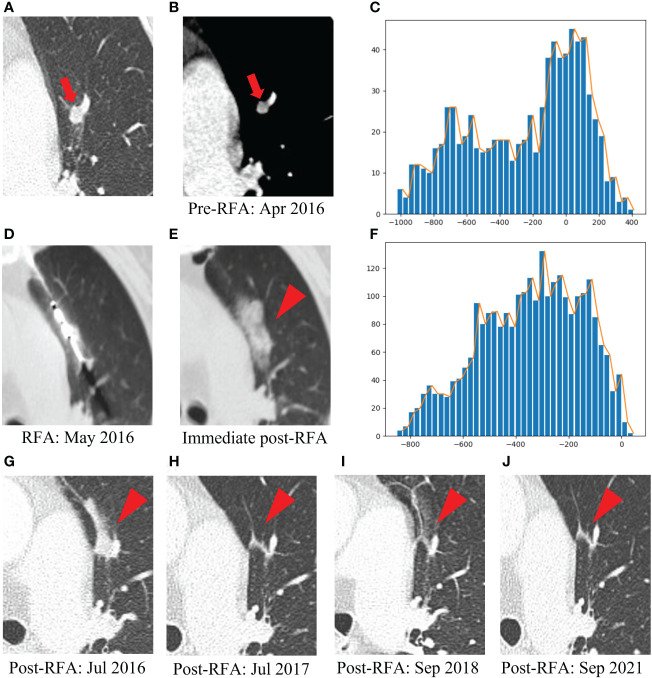
Example of a patient with post-lung RFA complete response. The multimodal data fusion model predicted the results: CR: 0.87, Non-CR: 0.13. **(A)** A 77-year-old female pT2N1M1R0 rectal cancer patient with a lung metastasis one year after rection, which was located in the left upper lobe (red arrow). **(B)** The mediastinal window of enhanced CT showed that the lesion was enhanced and adjacent to the blood vessel, with a maximum diameter of 6 mm (red arrow). **(C)** The histogram of the densities within this nodule on the pre-RFA CT scans displayed an asymmetric, skewed distribution corresponding to intra-tumoral enhancement (x-axis: attenuation in Hounsfield units, y-axis: number of voxels). **(D)** The RFA was performed under CT guidance. **(E)** IAH occurred after RFA (red arrowhead). **(F)** The histogram of the densities within the ablation zone on the immediate post-RFA CT scans was rather flat, without peak among the high tissular atenuations (x-axis: attenuation in Hounsfield units, y-axis: number of voxels). **(G)** Chest CT scan showed high density patch shadow in the ablation area one month after RFA (red arrowhead). **(H–J)** One year, two years and five years after RFA, chest CT scans showed that the lesion disappeared (red arrowhead). RFA, radiofrequency ablation; CR, complete response; IAH, intra-alveolar hemorrhage.

**Figure 7:**
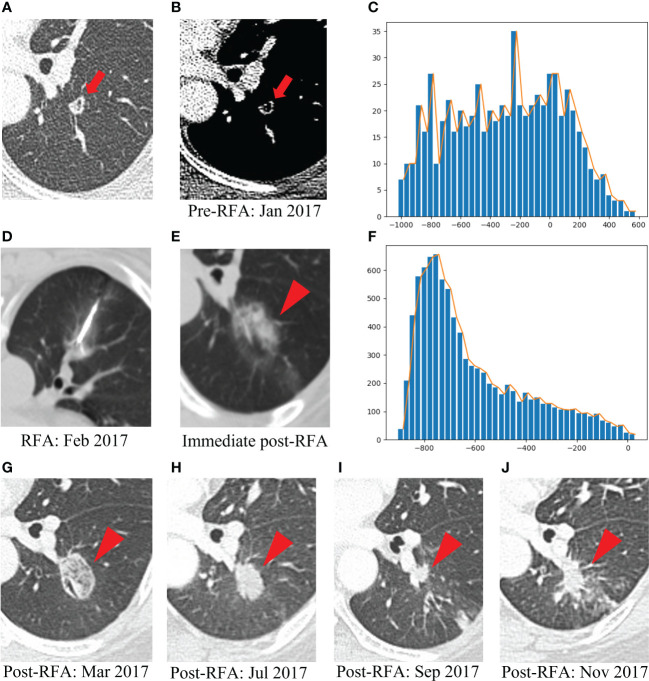
Example of a patient with post-lung RFA non-CR. The multimodal data fusion model predicted the results: CR: 0.06, Non-CR: 0.94. **(A)** A 52-year-old female pT4N1M1R0 rectal cancer patient with a lung metastasis eight months after rection, which was located in the left lower lobe (red arrow). **(B)** The mediastinal window of enhanced CT showed that the lesion was accompanied by small cavities with a maximum diameter of 9 mm (red arrow). **(C)** The histogram of the densities within this nodule on the pre-RFA CT scans was flat, without peak among the high tissular attenuations (x-axis: attenuation in Hounsfield units, y-axis: number of voxels). **(D)** The RFA was performed under CT guidance. **(E)** GGO occurred after RFA (red arrowhead). **(F)** The histogram of the densities within the ablation zone on the immediate post-RFA CT scans displayed an asymmetric, skewed distribution (x-axis: attenuation in Hounsfield units, y-axis: number of voxels). **(G)** Chest CT scan showed high density GGO with clear boundary one month after RFA (red arrowhead). **(H)** Five months after RFA, the GGO became a high-density nodule (red arrowhead). **(I)** Seven months after RFA, the high-density nodule shrank, but there was an irregular nodule near the vessel in the ablation area (red arrowhead). **(J)** Nine months after RFA, the irregular nodule was progressively enlarged and the recurrence was considered (red arrowhead). RFA, radiofrequency ablation; CR, complete response; GGO, ground glass opacity.

**Figure 6** presents one example of a patient with post-lung RFA CR, with a nodule in contact with a vessel, complicated by IAH. In contrast, **Figure 7** illustrates another example with post-lung RFA non-CR.

## Discussion

In 2016, the ESMO proposed a toolbox for oligometastases of CRC, which emphasized the clinical value of local therapy (38). In patients who are not eligible for surgery, RFA seems to have more evidence as a locoregional alternative for tumors< 3 cm (6).

After ablation, lung tumors undergo a natural evolution of the outcome process: in the early stage (within 1 week), the lesions are covered by GGO, with larger scopes than the lesions, and the interior of the lesions presents a low-density honeycomb appearance. In the middle stage (1 week to 2 months), the ablation area becomes larger, and an enhanced ring appears due to the absorption of inflammation around the lesion. Finally, in the late stage (after 2 months), the ablation area remains relatively stable or slightly larger, gradually shrinking or stabilizing after 6 months (24, 25). Therefore, contrast-enhanced CT of the chest at least 6 months after RFA was used to evaluate the efficacy of RFA in this study, so as to determine whether the lesions were completely ablated.

We found that the level of CA19-9 and location of the metastases were significant correlations with complete ablation. In terms of recurrence and survival prognosis, the combined evaluation of CEA and CA19-9 could obtain more relevant information than the evaluation of CEA alone (68, 69). However, this study found no significant association between CEA levels and complete ablation based on the multivariate logistic regression analysis. On the other hand, the location of nodules, including lung lower lobes, was an independent risk factor with values of OR > 2, possibly due to the influence of patient’s respiratory movement on the correct positioning of the probe. Also, IAH was associated with a higher risk of local recurrence, which reached significance in the univariate analysis, likely because of the increasing difficulties in locating the target nodule in the background of the dense and radiopaque zone. In addition, the heat sink effect was associated with a higher risk of incomplete ablation for tumors with blood vessel contact resulting from the blood flow and microscopic extension (12, 70, 71). However, our variables relating to vessels did not reach significance, probably with the influence caused by the enrolled cases close to the mediastinum.

In contrast to conventional CT-based imaging features, radiomics analysis enables a greater degree of information reflecting underlying biologic heterogeneity to be derived and qualified at a low cost (27, 46). A radiomics signature, as a panel of multiple features, has been regarded as a more powerful prognostic biomarker, which could provide additional information to clinical data, and has reportedly been a significant predictor for clinically relevant factors (72–74). Previous studies have demonstrated that the size and shape of metastases are the important risk factors for local recurrence (9, 12, 75), as the shape feature selected by MRMRA. In addition, GLRLM features could quantify gray level runs, defined as the length in a number of consecutive pixels that have the same gray level value (76) and could reflect the volumetric texture of the early ablation zone (77). A GLCM feature, which could reflect and quantify homogeneity to reflect the risk of local recurrence, is a common method of describing texture by studying the spatial correlation characteristics of gray levels (78). A GLDM feature quantifies gray level dependencies, which correspond the number of connected voxels within distance δ dependent on the center voxel (79), likely reflecting the difficulties in identifying the nodule.

Recent work has highlighted important efficacy and prognostic information captured in radiological, clinicogenomic, and histopathological data, which can be exploited through machine learning. However, little is known about the capacity of combining features from these disparate sources to improve the prediction of treatment response. Therefore, we combined radiomics with patients’ clinical variables to construct multimodal data fusion models to objectively and accurately evaluate the immediate efficacy of RFA for CRC lung metastases.

An observer study was conducted by testing an independent dataset to validate the performance of models (i.e., results shown in **Tables 4**, **5** and **Figure 5**). Compared with the baseline model only based on clinical variables, the radiomics-based models showed further improvement in performance with a significant statistical difference (P<0.05). Compared with the model only based on radiomics features, the corresponding performance indicators of the multimodal data fusion model (Radiomics + Clinical) were higher, but the Delong test did not confirm significant difference (P>0.05) between the models, indicating that the radiomics features have a dominant role in the models. At the same time, it suggests that the clinical variables could provide supplementary information to improve the predictive performance of the models, although they could not reach significance, possibly because of the limited sample size.

The main advantages of this study are as follows: first of all, different types of data might contain complementary information; therefore, we developed novel multimodal data fusion models integrating radiomics features based on radiological data and clinical variables originating from textual data for evaluating early ablation efficacy. In the second place, we proposed an information fusion scheme based on preoperative and immediately postoperative CT images, which could integrate the characteristics of the same target area in different periods. Finally, we adopted the GMM method (80) proposed in the previous study to conduct more reasonable data division to improve the model’s stability, accuracy and generalization, and minimize the deviation problem resulting from limited sample size when training the model.

There are few studies on the application of artificial intelligence methods to evaluate the efficacy of radiofrequency ablation for CRC lung metastases. A recent study (81) has retrospectively observed the instantaneous changes in intratumor density heterogeneity after MWA of pulmonary tumors *via* radiomics-based CT features and determined the prognostic value in predicting treatment response and local tumor progression (LTP). However, only 50 patients with different diseases (39 primary and 11 metastatic) were enrolled, which could not guarantee a sufficient sample size and the homogeneity of disease. In addition, it was not appropriate to evaluate ablation efficacy by chest contrast-enhanced CT afterablation, which was usually used as the baseline for evaluation (82). Another retrospective study (20) utilized radiomics, clinical, radiological, and technical features to access local control of 48 CRC patients with 119 lung metastases treated by RFA. In order to observe the nodule position in the ablation zone (categorized as nodule seen and remote from borders, or not [i.e., hidden or marginal]), patients underwent chest CT 48 hours after RFA. However, the related results might be subjective among doctors because of different experiences, so they could not assist operators in evaluating the ablation efficacy during the operation, thus allowing for more timely interventions, and in turn, reducing tumor load and prolonging overall survival (83).

Despite the promising results, our study has several limitations. Firstly, the sample size was relatively small because of strict exclusion criteria regarding imaging follow-up. Secondly, the immediate chest CT after RFA was a non-contrast-enhanced CT which might result in the loss of some potentially valuable information related to efficacy. Thirdly, the absent of deep learning algorithm which could identify non-specific features of target lesions and surrounding tissues through automatic learning to achieve information complementation. Thus, a larger patient population from multicenter with deep learning algorithm might further improve the performance in future studies.

In conclusion, the novel multimodal data fusion model (combining radiomics features and clinical variables) was developed to assess the early ablation efficacy. Based on these promising results, our study provides evidence that could assist interventional physicians in objectively and accurately evaluating the immediate efficacy of RFA for CRC lung metastases so as to make necessary supplementary treatment during operation.

## Data availability statement

The original contributions presented in the study are included in the article/supplementary material. Further inquiries can be directed to the corresponding authors.

## Ethics statement

The studies involving human participants were reviewed and approved by Fudan University Shanghai Cancer Center. The patients/participants provided their written informed consent to participate in this study. Written informed consent was obtained from the individual(s) for the publication of any potentially identifiable images or data included in this article.

## Author contributions

HH, DZ and HC: Methodology, Writing-Reviewing and Editing; CC and YiW: Data Collection and statistical analysis; HH and HC: Evaluation and Validation; DZ: Software and Models Building; LX, YaW and XH: Ablation Operations; WL and YY: Conceptualization and Supervision. All authors contributed to the article and approved the submitted version.
